# Ambulatory Same-Day Map-and-Treat Angiography for Selective Internal Radiation Therapy Using a Transradial Approach

**DOI:** 10.7759/cureus.27741

**Published:** 2022-08-07

**Authors:** Joshua P Frost, Jon Bell, Jeremy Lawrance, Pavan Najran, Damian Mullan

**Affiliations:** 1 Radiology and Interventional Radiology, The Christie NHS Foundation Trust, Manchester, GBR; 2 Interventional Radiology, The Christie NHS Foundation Trust, Manchester, GBR; 3 Radiology, The Christie NHS Foundation Trust, Manchester, GBR

**Keywords:** liver cancer-directed therapies, day-case procedures, radial artery access, liver metastases, selective internal radiation therapy (sirt)

## Abstract

Historically, selective internal radiation therapy (SIRT) with yttrium-90 (Y-90) requires a two-week interval between workup and treatment (map and treat). The intervening gap between workup and treatment is used to plan for the dose required and obtain delivery of the radioactive Y-90. During the coronavirus disease 2019 pandemic, the delivery of a robust SIRT service was challenging due to unprecedented demands on all hospital services. Emergent practice changes were required to ensure this service could still be delivered to patients while retaining sufficient inpatient hospital beds and services for acutely unwell patients.

In response to this, the interventional radiology team proposed the retention of a full SIRT service by removing the historical two-week interval between map and treat, delivering both components of the SIRT procedure on the same day. A traditional approach using femoral access would require a prolonged period of immobility and potentially an overnight stay. By adopting a transradial approach without sedo-analgesia, an ambulatory day-case map and treat SIRT with no post-procedure immobilisation was performed.

This case report demonstrates the technical feasibility of same-day ‘map-and-treat’ SIRT, highlighting a paradigm shift from the conventional femoral access method and immobilisation to an ‘ambulatory’ approach with immediate mobilisation post-procedure.

## Introduction

Selective internal radiation therapy (SIRT) is an established procedure for treating primary and secondary liver tumours, delivering yttrium 90 (Y-90) radioactive microbeads directly into the liver via the hepatic artery [1].

The SIRT procedure has two key stages, a workup or ‘mapping’ stage, and a subsequent treatment stage [2]. The purpose of the first stage is to map and define the hepatic arterial anatomy to identify anatomical variance and assess flow in the vessels. Historically, a second key component of the mapping procedure was to coil-occlude/embolise any hepatic or gastric branches that might complicate treatment, or lead to inadvertent Y-90 delivery outside of the liver [3]. The final component of the mapping procedure was to produce a trial run of the eventual Y-90 delivery by administrating a non-harmful, surrogate marker for Y-90, in this case, technetium-99 macro-aggregated albumin (^99m^Tc MAA).

After ^99m^Tc MAA delivery, the patient proceeds to a single photon emission computed tomography scan (SPECT) or planar SPECT scan to identify the sites of ^99m^Tc MAA uptake and to hopefully exclude extrahepatic uptake that might make it unsafe to proceed with the Y-90 delivery. More recently, the ^99m^Tc MAA scan has evolved from a basic surrogate to exclude extrahepatic uptake, becoming an important surrogate to estimate what the most effective dose of Y-90 might be for each individual patient; a concept of personalised dosimetry [4].

Following this, the patient would go on to have a separate angiogram for Y-90 delivery and treatment. Traditionally, SIRT has required a two-week interval between workup and treatment. The interval gap of two weeks allows for a review of the angiogram and SPECT images, planning of the dose of Y-90, and time to order and receive the Y-90, which usually arrives from an international manufacturer. The gap of two weeks also allows time for any deployed vascular coils to achieve full occlusion of the coiled blood vessel. However, there has been a trend over the last several years to move away from coiling blood vessels in SIRT mapping procedures, as it is felt that blocking some vessels might lead to altered flow dynamics elsewhere in the liver [5]. Thus, the need to wait for vessel occlusion is not as important a concept as it was several years ago. Globally, many SIRT procedures still have a variable interval between map and treat, but more recently, single-day SIRT map-and-treat procedures have been described [6].

Same-day map and treat for SIRT is feasible as manufacturers of Y-90 are now providing increasingly specialised options for tailoring doses specific to the patient on the day they are required. Sirtex Medical Ltd. has developed Y-90 resin microspheres (Sir-Spheres®, Sirtex Medical Ltd., Sydney, Australia) which enable a personalised dose draw at any time on the day of the procedure. Furthermore, Sirtex’s FLEXdose delivery programme provides versatility when ordering doses, as, depending on when the order was made, the desired activity can be achieved by adjusting the number of spheres delivered during SIRT. These factors permit ordering of the dose before evaluation of the ^99m^Tc MAA scan (the order, map, treat concept) and preparation of an appropriate patient-specific activity that occurs on the day of treatment. This ensures that both the mapping and treatment components of the procedure can be performed consecutively. However, there are some time pressures during a same-day map-and-treat procedure, and pre-SIRT imaging should be thoroughly reviewed to decide on a provisional treatment plan and estimated dose to help streamline the more exact dose estimation and delivery on the day.

Day-case map and treat has inherent advantages that have proven to be particularly relevant since the onset of the coronavirus disease 2019 (COVID-19) pandemic. During the pandemic, our hospital clinical advisory group (CAG) agreed to prioritise cancer treatments with curative intent, thus limiting other treatments to create the capacity to treat patients with COVID-19. Although SIRT can be delivered with curative intent, the majority of patients are treated palliatively, with the aim of achieving local disease control.

To mitigate this risk to our SIRT service, we wished to assess the technical feasibility of an ambulatory single-day ‘map-and-treat’ protocol, incorporating both the workup procedure (^99m^Tc MAA dosimetry) and Y-90 treatment. Our aim was to reduce the impact on hospital resources by limiting the number of hospital admissions and overnight stays, improving patient mobility, and eliminating the risk of being unable to administer the Y-90 on a subsequent day should COVID-19 escalate. For this practice to be attainable, we opted to perform the procedure using transradial access as opposed to femoral access. Cardiologists, and, more recently, radiologists, have adopted a radial approach to address morbidity from femoral puncture, and specifically to assist with same-day discharge, allowing for early ambulation and reduced aftercare [7-9]. Transradial access has several advantages over femoral access. It is associated with a lower haemorrhage risk, a shorter length of hospital stay, a shorter period of immobilisation, lower costs, and greater levels of patient satisfaction than with transfemoral access [10].

Additionally, femoral puncture post-procedure care relies mainly on bed rest, with patients having to remain supine for up to four hours post-catheter removal to mitigate the risk of bleeding (e.g. retroperitoneal haemorrhage), bruising, and pseudoaneurysm formation [11]. Though vascular occlusion devices are an option to facilitate more immediate haemostasis at the puncture site, they are not ideal in the setting of performing SIRT as a day-case procedure, where access following the initial ‘mapping’ angiogram is needed in a matter of hours for the ‘treatment’ angiogram.

With these points in consideration, this case report outlines the paradigm shift and feasibility of same-day ‘map-and-treat’ SIRT, describing the process with resin microspheres, and how this may continue to be a viable option for patients in the future.

## Case presentation

**Figure 1:**
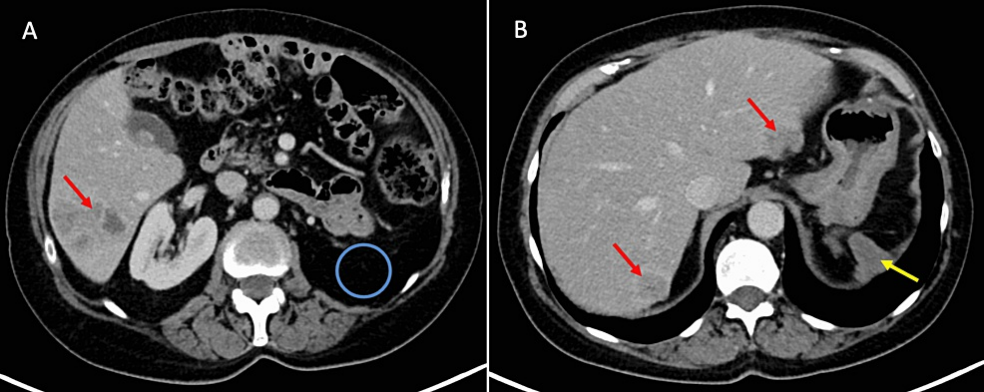
Portal-phase CT of the abdomen prior to SIRT therapy. (A) Segment 6 liver metastasis (red arrow) with an absence of the left kidney in the left renal bed (blue circle) after left adrenalectomy and nephrectomy. (B) Segment 2 and segment 7 liver metastases (red arrows). A haematoma of the resection bed (yellow arrow) was present. This was deemed stable with subsequent imaging. CT: computed tomography; SIRT: selective internal radiation therapy

A 75-year-old female with resected adrenocortical carcinoma and subsequent liver metastases (Figure 1) was selected for treatment with SIRT following tumour board consensus discussion. The patient lived 260 km from our institution and wanted to limit the number of hospital visits, particularly during the pandemic.

**Figure 2:**
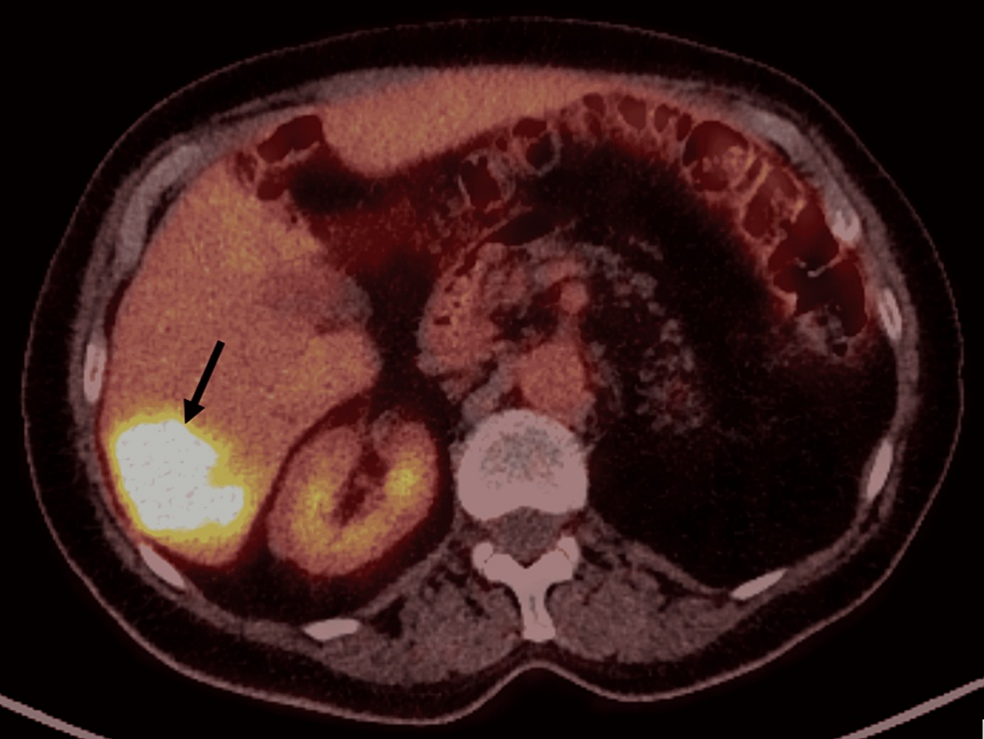
Outpatient PET-CT (pre-SIRT). PET-CT performed pre-SIRT showing solid FDG avid tumour (black arrow) with an SUVmax of 18.6. PET-CT: positron emission tomography-computed tomography; SIRT: selective internal radiation therapy; FDG: fluorine-18-labelled fluorodeoxyglucose; SUVmax: maximum standardized uptake value

Outpatient positron emission tomography-computed tomography (PET-CT) with intravenous (IV) contrast (Figure 2) showed fluorine-18-labelled fluorodeoxyglucose (FDG)-positive liver metastases and confirmed no extrahepatic disease, with normal arterial anatomy (type I modified Michel’s classification) [12]. The plan was to deliver whole liver treatment with resin microspheres via two separate ‘lobar’ infusion sites. Tumour/normal liver volume was measured, planned activity calculated using the body surface area (BSA) method, and lobar division corresponding to Cantlie’s line.

Access to the common hepatic artery was via a radial artery approach. The main catheter was a 5-French BERN catheter (Impress® Diagnostic Peripheral Catheters, Merit Medical) and a 2.7-French microcatheter (Progreat™ Micro Catheter System, Terumo International Systems).

**Figure 3:**
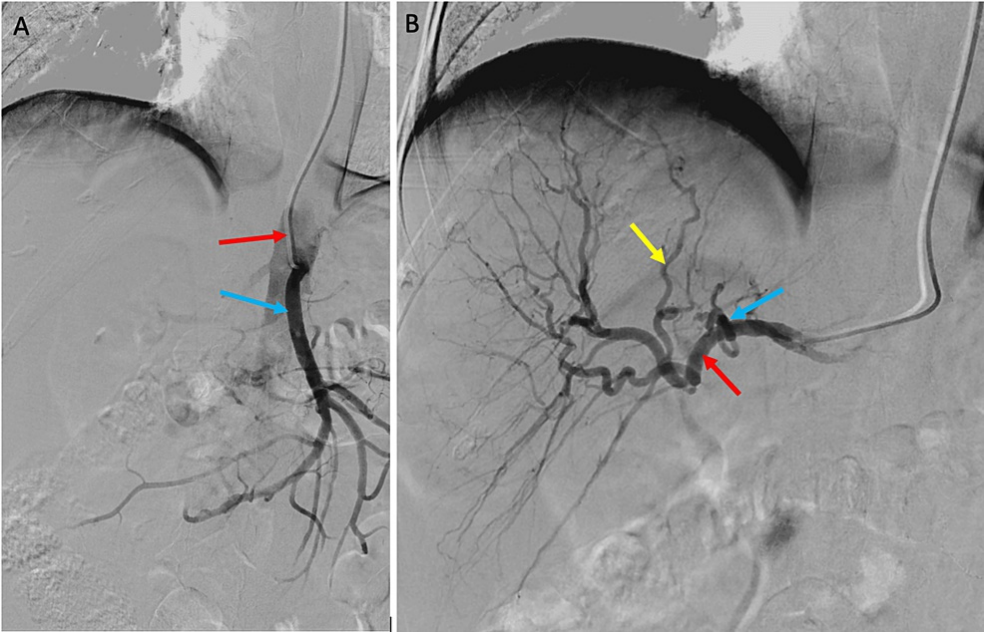
Fluoroscopic image (digital subtraction angiography) showing contrast injection through the arterial catheter. (A) Mesenteric angiogram demonstrating the catheter (red arrow) selecting the superior mesenteric artery (blue arrow) via a caudal approach from the radial artery, with no accessory branch or replaced supply to the liver. (B) Coeliac artery angiogram showing left (blue arrow) and right (red arrow) hepatic arteries. Segments 4 and 1 of the liver are being supplied from a right hepatic artery branch (yellow arrow).

**Figure 4:**
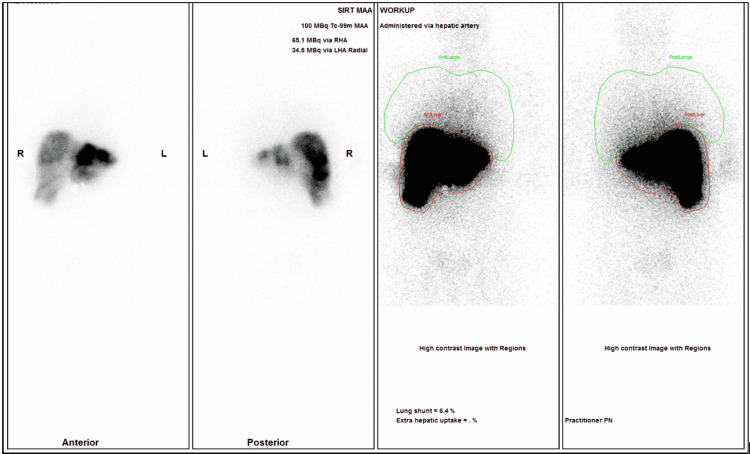
SPECT scan planar images following 99mTc MAA injection. SPECT scan showing no extrahepatic uptake of ^99m^Tc MAA, allowing same-day SIRT treatment to proceed. SPECT: single photon emission computed tomography; 99mTc MAA: technetium-99 macro-aggregated albumin; SIRT: selective internal radiation therapy

Angiography at 10:30 am demonstrated normal superior mesenteric artery anatomy (Figure 3A). A conventional left and right hepatic artery were seen to originate 1.5 cm distal to the gastroduodenal artery (GDA) (Figure 3B). Empiric coil embolisation of the GDA was thus not necessary. A cone beam CT (CBCT) performed with the micro-catheter tip beyond the origin of the left hepatic artery demonstrated that the right hepatic artery was supplying most of the liver, excluding segments 2 and 3, which were supplied by the left hepatic artery. ^99m^Tc MAA was administered at 12:05 pm. The patient was transferred for a SPECT-CT scan at 12:45 pm, confirming good tumour uptake, no extrahepatic uptake, and a 6.4% lung shunt (Figure 4). The nuclear medicine physician measured the perfused tumour and normal liver volumes based on angiosomes defined by CBCT. The activity of Y-90-resin microspheres was re-calculated by the medical physics team to achieve the required Y-90 split doses.

The patient returned to interventional radiology for the treatment angiogram at 2:30 pm, with resin microspheres delivered at 3:30 pm, approximately three and a half hours after the ^99m^Tc MAA injection (299 MBq into the left branch, and 1,423 MBq into the right). The subsequent uptake scan demonstrated good tumour uptake of Y-90 and no extrahepatic uptake. The patient had mild grade 1 symptoms relating to the post-embolisation syndrome, which were controlled with oral medications, and did not require re-admission.

**Figure 5:**
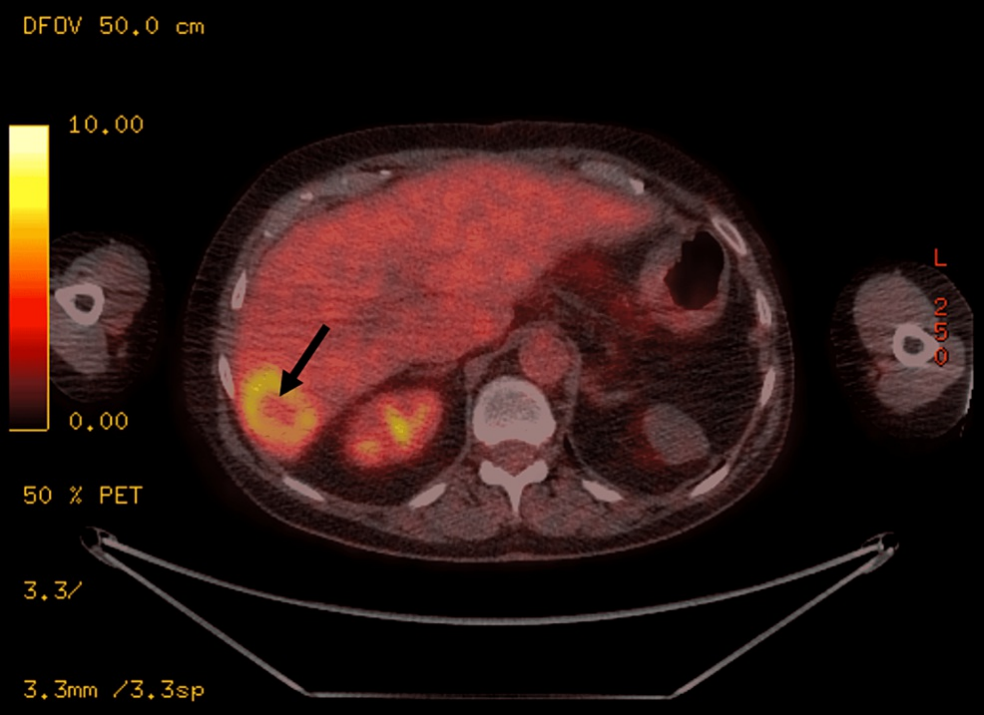
Outpatient PET-CT (post-SIRT). PET-CT three months post-SIRT treatment showing a reduction in the size of the segment 6 metastatic deposit from 5.5 cm to 3.5 cm. There was a reduction in the maximal FDG avidity to 8.4, with central necrosis present (black arrow). PET-CT: positron emission tomography-computed tomography; SIRT: selective internal radiation therapy

Follow-up PET-CT 12 weeks post-SIRT showed a reduction of all liver tumours with an associated reduction in FDG avidity (Figure 5).

## Discussion

​​​​​​With a fully engaged multidisciplinary team, we demonstrated that same-day map-and-treat SIRT is achievable and safe. When aligned with radial access, it can become truly ambulatory, conferring advantages by reducing patient travel, preventing two overnight hospital admissions, reducing patient exposure to staff, and allowing immediate ambulation and self-care. It also removed the inherent risk associated with a conventional SIRT with a two-week gap whereby patients might undergo the first-stage workup, but then fail to proceed to second-stage treatment if COVID-19 contingency plans were escalated at our hospital.

There are also some technical points to consider. There is a theoretical additional embolic effect from the temporally close administration of ^99m^Tc MAA and Y-90 resin microspheres. With the preparation of^ 99m^Tc MAA used (Pulmocis®) it was determined that approximately 0.6-1.2 million microparticles of MAA would be administered in the workup. The question remains of what embolic effect may be caused by the presence of these remaining ^99m^Tc MAA microparticles by the time of treatment with up to 44 million Y-90 resin microspheres. Although there is no clinical data confirming the time for complete absorption or elimination of ^99m^Tc MAA in the liver, the pharmacokinetics of MAA in the lungs from perfusion studies show that elimination depends on the size of MAA [13]. Considering a variable size between 5 to 140 microns, the time for complete absorption in the lung varies from two to eight hours. If a median size of 60-80 microns is assumed, this time could be around four to five hours, but this may not be clinically transferrable to ^99m^Tc MAA absorption in the liver. Based upon the drug kinetics of ^99m^Tc MAA, a biological half-life of three hours has been reported in normal liver tissue, giving an effective half-life of two to three hours post-administration. In practice, two to three hours will have passed by the time the patient leaves the angiography suite to attend the ^99m^Tc MAA scan and returns after methodical planning of the treatment.

In the case described, radial access was used for both the mapping and treatment components of SIRT. Radial access can be performed either at the distal styloid (proximal radial access) or distally at the snuff box [14]. We opted for a distal left radial artery/anatomical snuff box puncture for the workup, with a second conventional radial puncture for the treatment. The rationale for this was to reduce nursing aftercare [15], permit ambulation and self-care between procedures [16], and reduce portering requirements. A catheter could have been left in situ between the map and treat, but it was felt that this would require increased nursing support to assess/flush an indwelling sheath between the cases. Following the relaxation of rules, we now routinely use a radial artery catheter with a pressure transducer attachment to auto-flush the catheter between workup and treatment, preserving catheter hygiene and preventing a catheter clot.

At some centres, sedo-analgesia, including midazolam, is used during visceral angiography. It is also commonly used in transradial access to achieve anxiolysis and to reduce the incidence of radial artery spasms [16]. However, as midazolam’s sedating effects would limit the ability to provide an ‘ambulatory’ service, verapamil and fentanyl were used in this case to good effect. This allowed the patient to mobilise immediately after both the mapping and the treatment procedure, nullifying the need for immobilisation and monitoring of consciousness levels following sedation.

Overall, patient selection, assessment, and thorough informed consent are important to define who might be the best candidates anatomically, physiologically, and psychologically for this procedure.

## Conclusions

Ambulatory same-day ‘map and treat’ has been proven to be technically feasible, offering a number of distinct advantages to clinicians, hospitals, and patients. Several factors contributed to the success of this ambulatory approach.

First, a transradial access route conferred an advantage over femoral access by allowing immediate mobilisation post-procedure, reducing the need for nursing aftercare. Furthermore, the transradial procedures were performed without administering midazolam, facilitating earlier mobilisation by avoiding sedation. Finally, the use of Sirtex’s FLEXdose delivery programme using Y-90 resin microspheres (Sir-Spheres®, Sirtex Medical Ltd., Sydney, Australia) meant that the IR team could draw the appropriate patient-specific activity on the day of treatment, ensuring both the mapping and treatment angiograms could be performed on the same day.

However, we do acknowledge that logistical challenges are posed by completing a same-day map-and-treat SIRT (e.g. timely and accurate reporting of pre-SIRT imaging), and further research is also required to determine if the embolic effect of ^99m^Tc MAA and the short interval to resin microspheres delivery has a significant impact on flow dynamics and subsequent Y-90 dosimetry.
